# Delayed reinforcement of costimulation improves the efficacy of mRNA vaccines in mice

**DOI:** 10.1172/JCI183973

**Published:** 2024-10-21

**Authors:** Sarah Sanchez, Tanushree Dangi, Bakare Awakoaiye, Min Han Lew, Nahid Irani, Slim Fourati, Pablo Penaloza-MacMaster

**Affiliations:** 1 Department of Microbiology-Immunology, and; 2 Department of Medicine, Division of Allergy and Immunology, Feinberg School of Medicine and Center for Human Immunobiology, Northwestern University, Chicago, Illinois, USA.

**Keywords:** Immunology, Adaptive immunity

## Abstract

mRNA vaccines have demonstrated efficacy during the COVID-19 pandemic and are now being investigated for multiple diseases. However, concerns linger about the durability of immune responses, and the high incidence of breakthrough infections among vaccinated individuals highlights the need for improved mRNA vaccines. In this study, we investigated the effects of reinforcing costimulation via 4-1BB, a member of the TNF receptor superfamily, on immune responses elicited by mRNA vaccines. We first immunized mice with mRNA vaccines, followed by treatment with 4-1BB costimulatory antibodies to reinforce the 4-1BB pathway at different time points after vaccination. Consistent with prior studies, reinforcing 4-1BB costimulation on the day of vaccination did not result in a substantial improvement in vaccine responses. However, reinforcing 4-1BB costimulation on day 4 after vaccination, when 4-1BB expression levels were highest, resulted in a profound improvement in CD8^+^ T cell responses associated with enhanced protection against pathogen challenges. A similar clinical benefit was observed in a therapeutic cancer vaccine model. We also report time-dependent effects with OX40, another costimulatory molecule of the TNF receptor superfamily. These findings demonstrate that delayed reinforcement of costimulation may exert an immunologic benefit, providing insights for the development of more effective mRNA vaccines for infectious diseases and cancer.

## Abstract 2

The two-signal model suggests that antigen stimulation and costimulation should occur at the same time for optimal activation of T cell responses. However, a study by the Penaloza-MacMaster lab suggests that after mRNA vaccination, delayed reinforcement of 4-1BB costimulation results in superior CD8 T cell responses relative to early reinforcement of costimulation.

## Graphical abstract

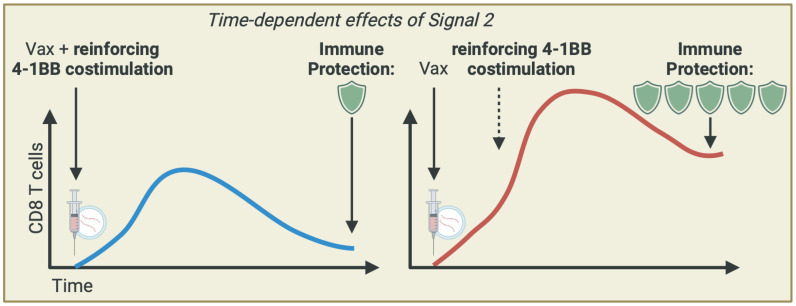

## Introduction

mRNA lipid nanoparticle (mRNA-LNP) vaccines are used to prevent severe SARS-CoV-2 infection and are being explored for multiple diseases such as influenza, HIV-1, and cancer (1–3). While mRNA vaccines have shown efficacy in preventing COVID-19, they do not always confer complete protection, as shown by the high incidence of breakthrough infections among vaccinated individuals, and their protection wanes over time, requiring multiple boosters. These limitations underscore the need to develop improved mRNA vaccine regimens. After vaccination, T cells play a critical role in rapidly clearing infected cells, and prior studies have shown that T cell responses are associated with reduced disease severity following breakthrough SARS-CoV-2 infection (4–6). According to the classical 2-signal model first described by Bretscher and colleagues, T cell responses are dependent on 2 concurrent signals; naive T cells must recognize their cognate antigen via their T cell receptor, and at the same time, they must receive costimulation (7). This model has been instrumental to understand how adaptive immune responses are generated and has had broad implications for the development of immunotherapies and vaccine adjuvants to reinforce costimulation at the time of priming. This model has also motivated the use of costimulatory regimens (e.g., costimulatory antibodies) to improve T cell responses after vaccination, but limited efficacy has been reported (8–12).

The most widely studied costimulatory pathway is CD28/B7, and the effects of other costimulatory pathways like 4-1BB/4-1BBL remain poorly understood. 4-1BB (also known as CD137) is a costimulatory receptor member of the TNF receptor superfamily (TNFRSF) and plays a role in T cell responses. 4-1BB is highly expressed on T cells and NK cells, among other cells, whereas its ligand (4-1BBL) is expressed mostly on antigen-presenting cells (13, 14). 4-1BB costimulation is important for effector T cell responses following bacterial and viral infections, and triggering of this pathway results in increased T cell proliferation, survival, and effector functions (10, 15–18). Prior studies have shown that 4-1BB costimulation renders T cells resistant to suppression by T regulatory cells (19), and due to its immunostimulatory effects, costimulatory anti–4-1BB antibodies (α4-1BB) have also been explored in cancer immunotherapy and for the treatment of chronic infection (16, 20–23). However, the effects of reinforcing 4-1BB costimulation on vaccine responses remain incompletely understood, with some reports showing detrimental effects (8–12). Here, we studied the effects of 4-1BB costimulation on immune responses elicited by mRNA vaccines. Like prior studies, we show that triggering 4-1BB costimulation on the day of mRNA vaccination does not significantly improve vaccine responses. However, we show that triggering 4-1BB costimulation on day 4 after vaccination, the time of maximal 4-1BB expression, significantly improves the efficacy of mRNA vaccines, rendering these vaccines more protective against breakthrough infections. These studies highlight a strategy to improve mRNA vaccines via time-dependent modulation of 4-1BB and suggest potential benefits of delaying 4-1BB costimulation for optimal CD8^+^ T cell expansion.

## Results

### Delayed 4-1BB costimulation induces a significant improvement in vaccine-elicited CD8^+^ T cells.

**Figure 1:**
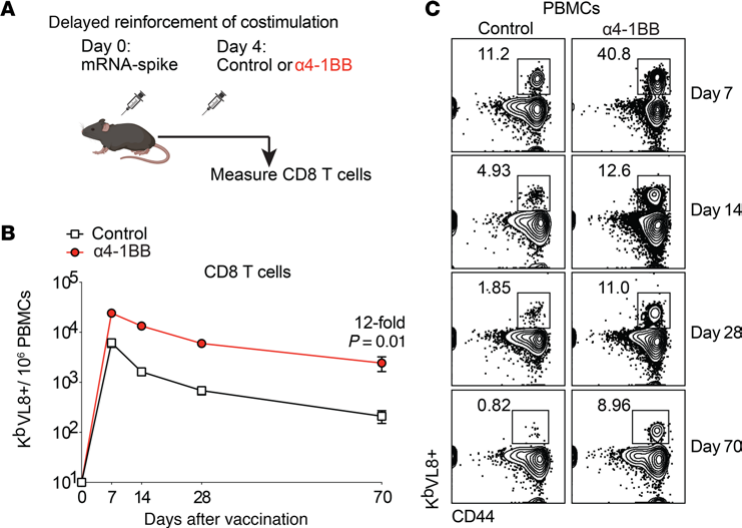
Reinforcing 4-1BB costimulation on day 4 after vaccination increases the number and durability of CD8^+^ T cell responses. (**A**) Experimental outline for evaluating whether treatment with α4-1BB on day 4 improves immune responses. Mice were immunized with 3 μg of an mRNA-spike vaccine followed by treatment with 50 μg of α4-1BB or control antibodies on day 4. (**B**) Summary of virus-specific CD8^+^ T cells. (**C**) Representative FACS plots of virus-specific CD8^+^ T cells. Data are from PBMCs. K^b^VL8 (shown in the *y* axis) is an MHC I tetramer used to detect SARS-CoV-2 spike–specific CD8^+^ T cells. Data are from 1 experiment, *n* = 4–5 per group/experiment; experiment was performed twice with similar results. Indicated *P* value in **B** was calculated with the Mann-Whitney test at the last time point.

Antigen recognition and costimulation are 2 indispensable signals needed for T cell responses, as the absence of costimulation results in anergy (24). While conventional wisdom from the classical 2-signal model is that antigen recognition and costimulation should occur simultaneously, the optimal timing of costimulation after antigen recognition remains unclear. We conducted experiments to investigate how the timing of 4-1BB costimulation affects immune responses after mRNA vaccination. We primed C57BL/6 mice intramuscularly with an mRNA vaccine expressing the SARS-CoV-2 spike protein (mRNA-spike) similar to the Moderna and Pfizer-BioNTech vaccines, and on the same day, we treated these mice intraperitoneally with costimulatory α4-1BB or control antibodies to examine the effect of reinforcing 4-1BB costimulation during the early priming phase (Supplemental Figure 1A; supplemental material available online with this article; https://doi.org/10.1172/JCI183973DS1). Reinforcing 4-1BB costimulation during the early priming phase did not improve CD8^+^ T cell responses relative to control (Supplemental Figure 1B) and exerted a negative effect on antibody responses (Supplemental Figure 1C). Like many other costimulatory receptors, 4-1BB is an activation-induced molecule (25), motivating us to examine whether reinforcing 4-1BB costimulation later during the immune response would improve vaccine responses (Figure 1A). Interestingly, treatment with costimulatory α4-1BB on day 4 after vaccination resulted in a potent and durable increase in CD8^+^ T cell responses in blood (Figure 1, B and C) and tissues (Supplemental Figure 2, A–C). This potentiation of CD8^+^ T cell responses was associated with higher Ki67 and PD-1 expression, suggesting enhanced proliferation and activation (Supplemental Figure 2, D and E). Treatment with costimulatory α4-1BB on day 4 after vaccination also improved degranulation and cytokine expression capacity on virus-specific CD8^+^ T cells (Supplemental Figure 2, F–I) and CD4^+^ T cells (Supplemental Figure 2J). There were no significant differences in the frequencies of short-lived effector cells and memory precursor effector cells (Supplemental Figure 2, K and L).

In addition, treatment with costimulatory α4-1BB on day 4 after vaccination resulted in a significant increase in systemic cytokines, especially GM-CSF, relative to control vaccination (Supplemental Figure 3A). Although GM-CSF was significantly upregulated after α4-1BB treatment, GM-CSF blockade did not abrogate the positive effect of α4-1BB on vaccine-elicited CD8^+^ T cells (Supplemental Figure 3, B and C). Reinforcing 4-1BB costimulation also upregulated IFN-γ (Supplemental Figure 3A), which is known to downmodulate mRNA protein translation (26). Therefore, we interrogated whether treatment with α4-1BB could reduce antigen expression after mRNA vaccination. To investigate this possibility, we measured antigen expression using an mRNA-LNP encoding a luciferase reporter. Our data show that α4-1BB treatment did not significantly alter antigen expression following mRNA vaccination (Supplemental Figure 4).

In the studies above, we administered a single low dose of costimulatory α4-1BB (50 μg) that was previously titrated to saturate all 4-1BB receptors for 3 days (16). Higher and repetitive doses of costimulatory α4-1BB (200 μg on days 4, 7, and 10) did not further improve CD8^+^ T cell responses relative to single treatment on day 4 (Supplemental Figure 5, A and B). Administration of costimulatory α4-1BB on day 4 did not significantly affect antibody responses after mRNA vaccination (Supplemental Figure 5C). Moreover, treatment with costimulatory α4-1BB 2 weeks after vaccination (contraction phase) did not affect immune responses (Supplemental Figure 6), demonstrating that the positive effects of costimulatory α4-1BB were time dependent. Altogether, these results show that day 4 was a critical time point to costimulate CD8^+^ T cells via 4-1BB, and further costimulation on later days did not confer an additional benefit.

### Effects of 4-1BB costimulation on CD8^+^ T cell differentiation.

**Figure 2:**
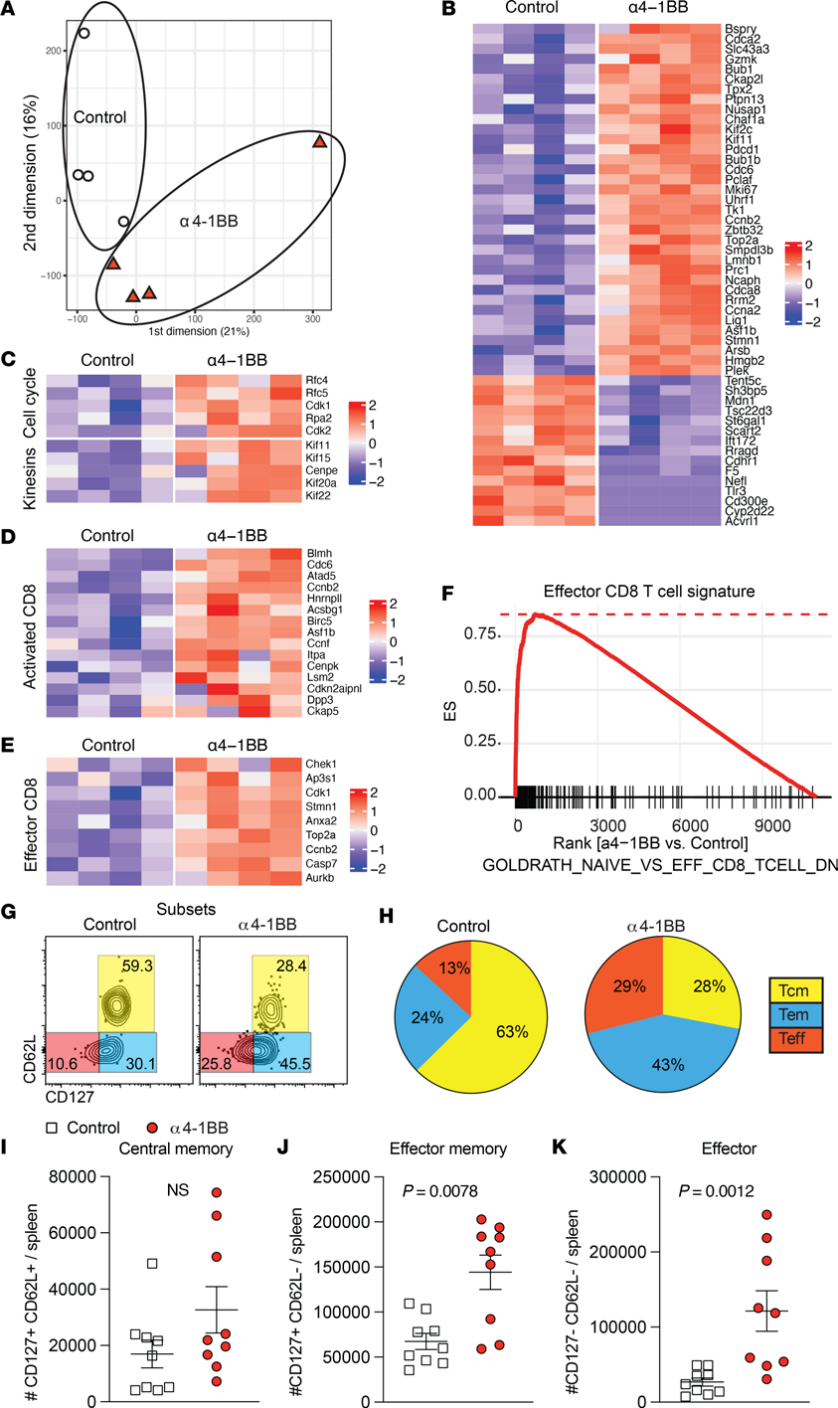
CD8^+^ T cell subset differentiation after reinforcing 4-1BB costimulation. Experimental outline was similar to that in Figure 1A. On day 7 after vaccination, splenic CD8^+^ T cells were MACS sorted. Subsequently, live CD8^+^CD44^+^K^b^VL8 tetramer^+^ cells were FACS-purified to approximately 99% purity and used for bulk RNA-seq. (**A**) PCA shows transcriptional clustering. (**B**) Heatmap showing row-standardized expression of selected proliferation and apoptotic genes. (**C**) Heatmap showing row-standardized expression of selected cell cycle (top) and kinesins (bottom) genes. (**D**) Heatmap showing row-standardized expression of selected activation genes. (**E**) Heatmap showing row-standardized expression of selected effector genes. (**F**) GSEA plot showing enrichment of effector genes. (**G**) Validation of gene expression results at the protein level. Representative FACS plots showing the frequencies of virus-specific CD8^+^ T cells (K^b^VL8^+^) that differentiate into effector, effector memory, and central memory T cell subsets. (**H**) Pie diagrams showing CD8^+^ T cell subsets. (**I**–**K**) Numbers of central memory, effector memory, and effector CD8^+^ T cells. All data are from tetramer^+^ (K^b^VL8^+^) cells from spleen. RNA-seq data are from 1 experiment, with *n* = 4 per group. Data in panel **H** are from 1 representative experiment, with *n* = 4 per group; the experiment was performed twice with similar results. All other data are from 2 experiments, with *n* = 4–5 per group/experiment. Indicated *P* values in **I**–**K** were calculated by the Mann-Whitney test.

Following an initial antigen encounter, CD8^+^ T cells differentiate into distinct subsets, including effector, effector memory, and central memory T cells (27–29). To examine whether reinforcing 4-1BB costimulation selectively favored the differentiation of specific subsets, we FACS-isolated splenic virus-specific CD8^+^ T cells on day 7 after vaccination and performed RNA-sequencing (RNA-seq) analyses. By principal component analyses (PCA), virus-specific CD8^+^ T cells clustered differently, suggesting transcriptional differences (Figure 2A). We observed enrichment of genes associated with cell proliferation, activation, and effector differentiation (Figure 2, B–E), and more pronounced effector signatures by gene set enrichment analyses (GSEA) (Figure 2F) in mice that received costimulatory α4-1BB on day 4. We validated these gene expression results at the protein level using flow cytometry. Consistent with the gene expression data, virus-specific CD8^+^ T cells after 4-1BB costimulation exhibited more pronounced effector (CD62L−CD127−) and effector memory (CD62L−CD127+) differentiation (Figure 2, G and H). There was a pattern of increased central memory CD8^+^ T cells (CD62L+CD127+) in mice that received α4-1BB, but the differences were not statistically significant relative to control (Figure 2I). Effector and effector memory CD8^+^ T cells were significantly greater in mice that received α4-1BB, relative to control (Figure 2, J and K).

### Generalizability to other vaccines.

**Figure 3:**
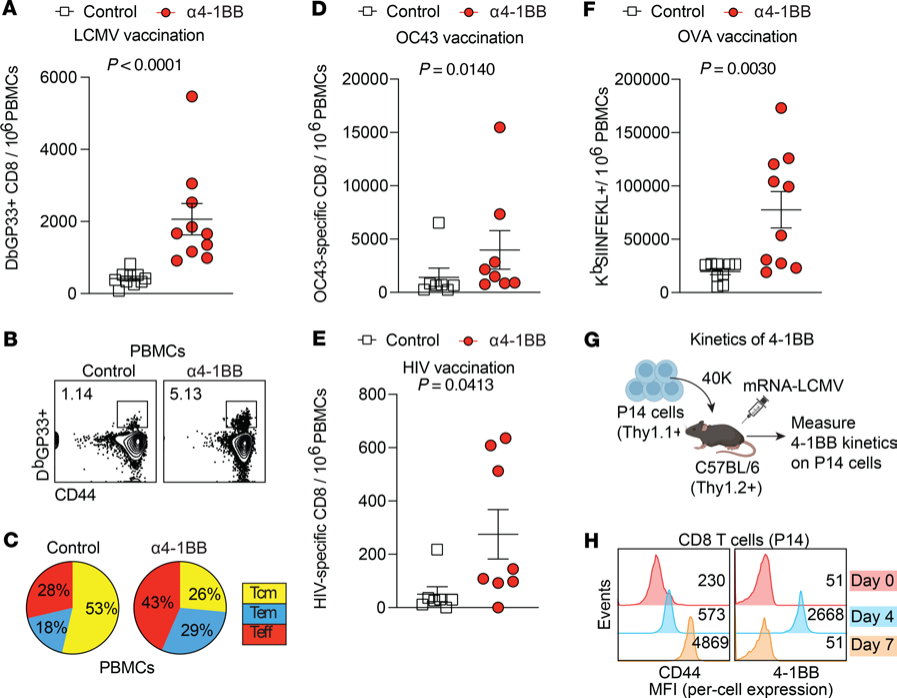
Generalizability to other mRNA vaccines. Mice were immunized with 3 μg of each respective mRNA vaccine followed by treatment with 50 μg of α4-1BB or control antibodies on day 4. (**A**) Summary of LCMV-specific CD8^+^ T cell responses. (**B**) Representative FACS plots of LCMV-specific CD8^+^ T cells. (**C**) Pie diagrams showing CD8^+^ T cell subsets (gated on LCMV-specific CD8^+^ T cells). (**D**) Summary of OC43 spike–specific CD8^+^ T cell responses. (**E**) Summary of HIV env–specific CD8^+^ T cell responses. (**F**) Summary of OVA-specific CD8^+^ T cell responses. Data from **A**–**C** and **F** are after tetramer staining; data from **D** and **E** are after intracellular cytokine stimulation using overlapping peptide pools (IFN-γ^+^). Data from **A**–**F** are from day 14 after vaccination, and are from 2 experiments, one with *n* = 5 per group/experiment and one with *n* = 2–5 per group/experiment. (**G**) Experimental outline for measuring 4-1BB following mRNA vaccination. P14 cells were transferred into C57BL/6 mice. One day after transfer, recipient mice were immunized with 3 μg of an mRNA-LCMV GP vaccine, and 4-1BB was measured on P14 cells at various time points. (**H**) 4-1BB on P14 cells after mRNA vaccination. Representative histograms showing 4-1BB expression on P14 cells. We utilized this P14 chimera model using a high number of P14 cells to allow us to detect 4-1BB expression on virus-specific CD8^+^ T cells at hyperacute points; endogenous virus-specific CD8^+^ T cells cannot be detected at hyperacute time points due to their low precursor frequency. Mean fluorescence intensity (MFI) is indicated on the *x* axis to denote “per-cell expression” of 4-1BB. This adoptive transfer experiment was performed 2 times, with *n* = 3 per group, showing similar results (peak of 4-1BB expression on day 4 after vaccination). All data are shown. Indicated *P* values in **A** and **D**–**F** were calculated by the Mann-Whitney test.

We then interrogated the generalizability of our observations using other mRNA-based vaccines. Consistent with the prior data, 4-1BB costimulation on day 4 resulted in a significant improvement in CD8^+^ T cells following vaccination with an mRNA vaccine against lymphocytic choriomeningitis virus (LCMV) (Figure 3A), and most virus-specific CD8^+^ T cells exhibited an effector and effector memory phenotype (Figure 3, B and C). Reinforcing 4-1BB costimulation on day 4 after vaccination also resulted in a significant improvement in CD8^+^ T cells following immunization with other mRNA vaccines, including a common cold coronavirus (OC43) vaccine (Figure 3D), a human immunodeficiency virus (HIV-1) vaccine (Figure 3E), and an ovalbumin (OVA) vaccine (Figure 3F). No statistically significant differences were observed in CD4^+^ T cell and antibody responses (Supplemental Figure 7). These data with multiple mRNA vaccines suggest that delayed 4-1BB costimulation was beneficial for CD8^+^ T cell responses.

We validated these observations with a different vaccine platform, a poxvirus vector used in the clinically approved Mpox vaccine that is based on modified vaccinia Ankara (MVA). Consistent with our prior studies with mRNA vaccines, we also observed improvement in poxvirus-specific CD8^+^ T cell responses when mice were treated with costimulatory α4-1BB on day 4 after vaccination (Supplemental Figure 8, A and B). No difference was observed in poxvirus-specific antibody responses (Supplemental Figure 8C). Furthermore, we tested an MVA-vectored vaccine expressing the SARS-CoV spike antigen derived from the original coronavirus of 2004 (MVA-SARS-1 spike) (Supplemental Figure 8D). With this vaccine, we also observed improvement in CD8^+^ T cell responses, but no difference in antibodies when 4-1BB was reinforced on day 4 after vaccination (Supplemental Figure 8, E and F). Taken together, our data from multiple vaccine platforms show that 4-1BB costimulation on day 4 after vaccination results in an improvement in CD8^+^ T cell responses.

### Kinetics of 4-1BB on vaccine-elicited CD8^+^ T cells.

We interrogated whether the time-dependent effects of 4-1BB costimulation were linked to varying expression levels of 4-1BB on CD8^+^ T cells. To answer this question, we measured 4-1BB expression on virus-specific CD8^+^ T cells at various time points after vaccination to determine whether there was a direct association between 4-1BB levels and response to 4-1BB costimulation. Since virus-specific CD8^+^ T cells cannot be detected on day 4 after vaccination due to low precursor frequency, we utilized an adoptive transfer model using P14 cells, which allowed us to examine virus-specific CD8^+^ T cells at hyperacute time points (Figure 3G). Interestingly, 4-1BB expression exhibited “zig-zag” kinetics, peaking on day 4 and returning to baseline levels by day 7 after vaccination (Figure 3H). Altogether, our data suggest that triggering 4-1BB at the time of maximal 4-1BB expression was beneficial for CD8^+^ T cell responses.

### Reinforcing 4-1BB costimulation on day 4 after vaccination confers enhanced vaccine protection against antigen challenges.

**Figure 4:**
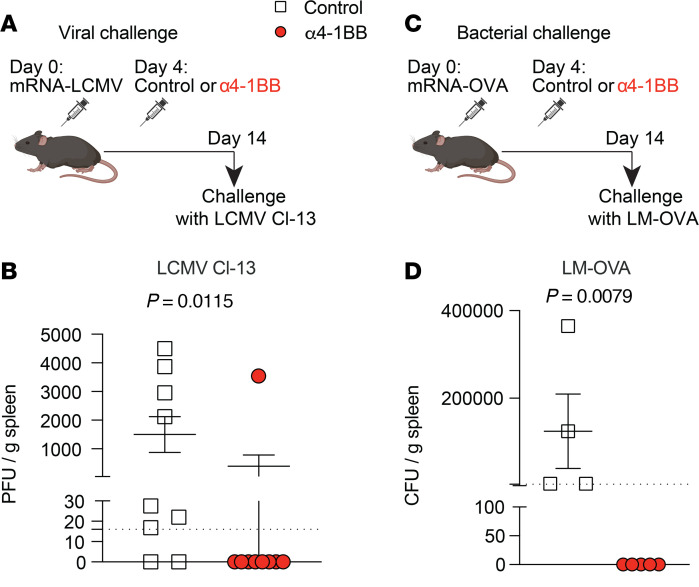
Reinforcing 4-1BB costimulation 4 days after mRNA vaccination induces sterilizing protection against pathogen challenges. (**A**) Experimental outline to examine whether treatment with α4-1BB on day 4 improves immune protection conferred by an mRNA-LCMV vaccine. (**B**) Summary of LCMV Cl-13 loads in the spleen on day 7 after challenge. On day 14 after vaccination, mice were challenged i.v. with LCMV Cl-13 (2 × 10^6^ PFU) and viral loads were quantified in Vero-E6 monolayers. (**C**) Experimental outline to examine whether treatment with α4-1BB on day 4 improves immune protection conferred by an mRNA-OVA vaccine. (**D**) Summary of LM-OVA bacterial loads in the spleen on day 3 after challenge. On day 14 after vaccination, mRNA-OVA–vaccinated mice were challenged i.v. with a supralethal dose of LM-OVA (1 × 10^7^ CFU) and bacterial loads were quantified in agar plates. In the challenge experiments, mice were immunized with 3 μg of the respective vaccine followed by treatment with 50 μg of α4-1BB or control antibodies on day 4. LCMV Cl-13 challenge data are from 2 experiments, one with *n* = 5 per group/experiment and one with *n* = 4 per group/experiment. Data from the LM-OVA challenge experiment are from one experiment, *n* = 4–5 per group. The control vaccines were still able to confer partial protection, relative to no vaccination (mean LCMV Cl-13 viral loads in unvaccinated mice = 1.3 × 10^7^ PFU/g; mean LM-OVA loads in unvaccinated mice = 1.1 × 10^6^ CFU/g). Indicated *P* values in **B** and **D** were calculated by the Mann-Whitney test.

SARS-CoV-2 vaccines confer robust protection in k18-hACE2 mice, making them unsuitable for examining differences in immune protection after 4-1BB costimulation. Thus, we utilized more stringent pathogen challenges to compare vaccine protection. We first vaccinated mice with an mRNA vaccine against LCMV and then treated these mice with α4-1BB or control antibodies on day 4 after vaccination (Figure 4A). After 2 weeks, we challenged these mice intravenously (i.v.) with a high dose of chronic LCMV Cl-13 and then measured weight loss and viral loads on day 7 after challenge (Figure 4A). Strikingly, most of the mice that received 4-1BB costimulation on day 4 after vaccination exhibited complete protection against this stringent arenavirus challenge (Figure 4B and Supplemental Figure 9). We validated these results with a different vaccine model, by vaccinating mice with an mRNA vaccine expressing OVA and then challenging them i.v. with *Listeria* *monocytogenes* expressing OVA (LM-OVA) (Figure 4C). Mice that received costimulatory α4-1BB exhibited complete protection upon challenge with a supralethal dose of LM-OVA (Figure 4D). These data suggested that delayed 4-1BB costimulation on day 4 after vaccination rendered the vaccines fully protective. Taken together, these results show that reinforcing 4-1BB on day 4 after vaccination, the time of maximal 4-1BB expression, potentiated CD8^+^ T cell responses and enabled them to more effectively protect the host upon subsequent pathogen challenges.

We also examined the effects of α4-1BB during a booster vaccination by re-administering these costimulatory antibodies after a booster vaccination (Supplemental Figure 10A). Following booster vaccination, there was a robust increase in recall CD8^+^ T cell responses in both groups and α4-1BB did not confer a significant benefit (Supplemental Figure 10B). These data suggest that memory CD8^+^ T cells may be less reliant on 4-1BB costimulation for their recall expansion, relative to naive CD8^+^ T cells. Another consideration is that costimulatory α4-1BB have been explored for various diseases, but their high toxicity profiles have precluded them from being licensed. Prior studies have shown that treatment with costimulatory α4-1BB can cause hepatotoxicity linked to increases in liver enzyme activity such as alanine aminotransferase (ALT) (25, 30), so we interrogated whether our low-dose α4-1BB regimen would induce a similar detrimental effect. With the single low dose tested in our vaccine studies (50 μg given on day 4 after prime), we did not observe upregulation of ALT activity relative to control, suggesting that this low-dose treatment was safe and well tolerated (Supplemental Figure 11).

**Figure 5:**
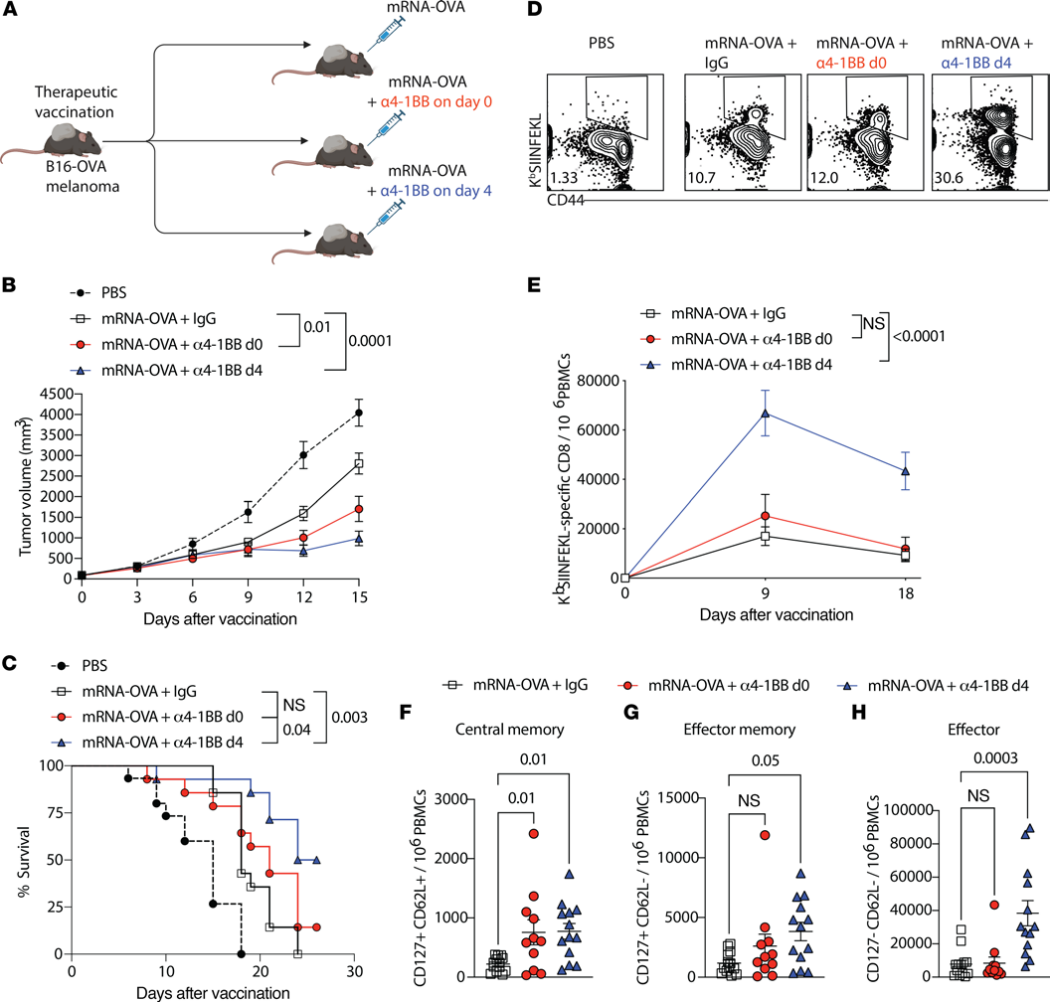
Delayed reinforcement of 4-1BB enhances the efficacy of a therapeutic cancer vaccine. (**A**) Experimental outline to examine whether treatment with α4-1BB on day 4 improves immune protection by a therapeutic cancer vaccine. Mice were challenged s.c. with 2 × 10^6^ B16-OVA tumor cells. On day 10 after tumor challenge, mice were vaccinated intramuscularly with 3 μg of mRNA-OVA. Mice received either control antibodies or α4-1BB (50 μg on day 0 or day 4 after mRNA-OVA vaccination). (**B**) Tumor control. (**C**) Survival. (**D**) Representative FACS plots showing CD8^+^ T cell responses on day 9 after vaccination. (**E**) Summary of OVA-specific CD8^+^ T cell responses on day 9. (**F**–**H**) Central memory, effector memory, and effector CD8^+^ T cells (K^b^SIINFEKL^+^ PBMCs) at 2 weeks after vaccination. Data are from 2 experiments, one with *n* = 6–7 per group and one with *n* = 8 per group. Indicated *P* value in **C** was calculated by the log-rank (Mantel-Cox) test; all other *P* values were calculated by 2-way ANOVA with the Holm-Šídák multiple-comparison test.

We performed additional experiments to interrogate whether delayed 4-1BB costimulation improves tumor control in a therapeutic cancer vaccine model. To answer this question, we first challenged mice subcutaneously with B16-OVA tumor cells. After the tumor was established, mice were immunized with an mRNA-OVA vaccine, and then treated with α4-1BB on day 0 or day 4 after vaccination to determine whether the timing of α4-1BB affected tumor control (Figure 5A). α4-1BB on day 0 after vaccination improved tumor control relative to vaccination alone, but the enhancement in tumor control was more significant when α4-1BB was administered on day 4 after vaccination (Figure 5B). Mice that received α4-1BB on day 4 after vaccination also exhibited improved survival relative to all groups (Figure 5C), and this was associated with enhanced CD8^+^ T cell responses (Figure 5, D and E), especially effector CD8^+^ T cell responses (*P* = 0.0003, Figure 5, F–H).

**Figure 6:**
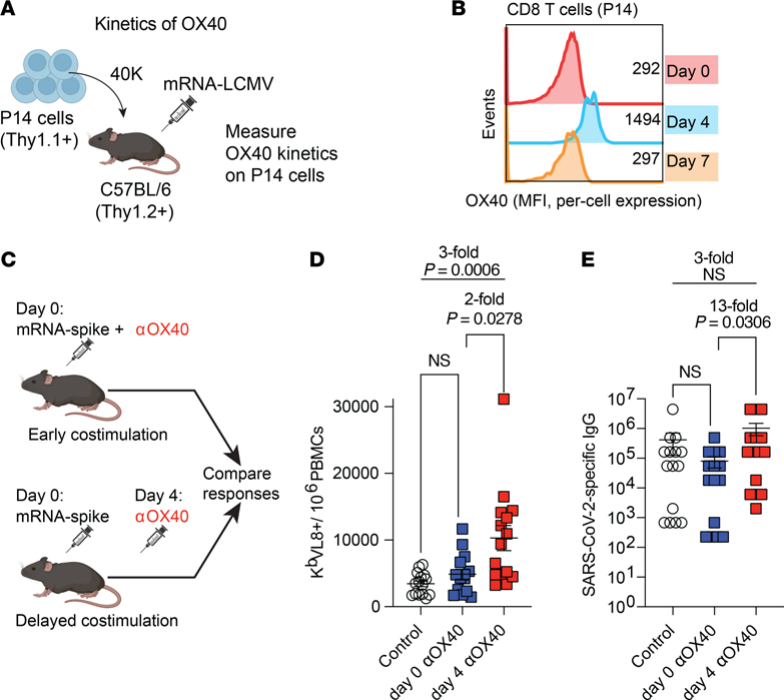
Generalizability to other costimulatory pathways: reinforcing OX40 costimulation on day 4 results in superior vaccine responses, relative to reinforcing OX40 costimulation on day 0. (**A**) Experimental outline for evaluating OX40 expression following mRNA vaccination. We utilized the same adoptive transfer model from Figure 3G. (**B**) Kinetics of OX40 on virus-specific CD8^+^ T cells after mRNA vaccination. This adoptive transfer experiment was performed 2 times, with *n* = 3 per group, showing similar results (peak of OX40 expression on day 4 after vaccination). (**C**) Time-dependent effects of OX40 costimulation following mRNA vaccination. Mice were immunized with 3 μg of mRNA-spike vaccine, followed by treatment with OX40 costimulatory antibodies (200 μg of αOX40, clone OX-86) on day 0 or day 4 after vaccination. CD8^+^ T cell responses (**D**) and antibody responses (**E**) on day 15 after vaccination are shown. Data in **D** and **E** are from 3 experiments, with *n* = 5 per group. Indicated *P* values in **D** and **E** were calculated by Kruskal-Wallis test with Dunn’s multiple-comparison test.

We then interrogated whether our observations could generalize to other costimulatory molecules. Interestingly, OX40, which is also a member of the TNFRSF, exhibited the same “zig-zag” kinetics as 4-1BB; its expression on virus-specific CD8^+^ T cells was highest on day 4 and returned to baseline levels by day 7 after vaccination (Figure 6, A and B). A prediction from these kinetics data is that the optimal time point for OX40 costimulation is day 4 after vaccination, since this time point corresponded to the peak expression of this molecule. To determine whether our prediction was correct, we immunized C57BL/6 mice with an mRNA vaccine, and on day 0 or day 4 after vaccination, we administered costimulatory αOX40 to reinforce OX40 costimulation at these different time points (Figure 6C). Reinforcing OX40 costimulation on the day of vaccination did not significantly improve CD8^+^ and CD4^+^ T cell responses, but reinforcing OX40 costimulation on day 4 resulted in a significant improvement in these responses (Figure 6D and Supplemental Figure 12). Moreover, reinforcing OX40 costimulation on day 4 resulted in superior antibody responses compared with reinforcing OX40 costimulation on day 0 (Figure 6E). Although there was a pattern of improved antibody responses with αOX40 on day 4, relative to control or α4-1BB on day 4, the difference was not statistically significant (Supplemental Figure 13).

## Discussion

mRNA vaccines have been administered to millions of people worldwide and have shown efficacy in preventing severe disease and death caused by SARS-CoV-2 infection. However, these vaccines do not confer complete protection and require multiple booster shots, underscoring the need for improved mRNA vaccines. In this study, we interrogated whether mRNA vaccines could be improved by reinforcing 4-1BB, a costimulatory molecule that is important for T cell activation. Costimulatory α4-1BB have been clinically tested in autoimmunity and cancer immunotherapy (22, 23), and the signaling molecules involved in 4-1BB costimulation are included in chimeric antigen receptor T cell therapies (31). Although 4-1BB is known to play a costimulatory role, there are reports of α4-1BB causing immunosuppression when delivered concomitantly with antigen. For example, a prior study reported that when costimulatory α4-1BB are administered at the time of LCMV infection, both T cell and antibody responses are impaired (32). Similarly, other studies have shown impaired immune responses when 4-1BB costimulation is reinforced at the time of vaccination, hindering the exploration of 4-1BB agonistic regimens as vaccine adjuvants (8, 9). The potentially detrimental effects of combining costimulation concurrently with antigen priming may not be exclusive to 4-1BB. Prior studies have shown that reinforcing CD40 or OX40 costimulation on the day of priming with LCMV leads to impaired immune responses (33, 34). Considering our data and those of others, we hypothesized that concurrent provision of Signal 1 and Signal 2 may not be optimal for vaccine-elicited immune responses and that temporally separating these signals may be necessary to fully unleash the immunostimulatory effects of costimulation.

Antigen recognition is metaphorically analogous to inserting the key to turn on a car, while costimulation is analogous to stepping on the accelerator. Employing this classical analogy, inserting the key and stepping on the accelerator at the same time can lead to “flooding of the engine.” This concept led us to hypothesize that extending the time interval between antigen recognition and costimulation would allow CD8^+^ T cells to “warm up” and upregulate their costimulatory receptors, rendering them more responsive to subsequent costimulation. Our kinetics data corroborate the inducible nature of 4-1BB following vaccination, and we observe that a temporal separation between vaccination and 4-1BB costimulation improves the protective efficacy of mRNA vaccines. In other words, such positive time-dependent effects could be explained by the fact that 4-1BB is an inducible costimulatory receptor (25). Thus, costimulatory α4-1BB may not engage many 4-1BB receptors on naive CD8^+^ T cells since these cells have not yet upregulated 4-1BB on their surface. However, treatment with costimulatory α4-1BB on day 4 (when CD8^+^ T cells express high levels of 4-1BB) may trigger more potent costimulatory signaling, leading to a more robust expansion of CD8^+^ T cells. The inducible nature of 4-1BB as well as other costimulatory receptors likely ensures the proper sequence of signaling events, precluding “out-of-order” signaling (e.g., costimulation preceding antigen recognition).

We also examined the specific CD8^+^ T cell subsets that were increased by α4-1BB. We show that α4-1BB on day 4 results in a significant increase in effector CD8^+^ T cells. These data are consistent with a prior study from the Watts laboratory showing that 4-1BB is important for the persistence of effector CD8^+^ T cells in tissues (35). A cardinal feature of effector and effector memory CD8^+^ T cells is their “response-ready” state (27), which provides rapid protection from breakthrough infection, but these cells may have a shorter lifespan than central memory CD8^+^ T cells. Notwithstanding the lower durability of effector memory CD8^+^ T cells relative to central memory CD8^+^ T cells, we detected increased CD8^+^ T cell responses after 2 months of vaccination in mice that received costimulatory α4-1BB, suggesting long-term enhancement of responses by 4-1BB costimulation. Future studies will examine the durability of CD8^+^ T cell responses over longer periods of time.

The complete protection observed in the chronic LCMV challenge and *Listeria* challenge models is likely not only due to a numerical increase in antigen-specific CD8^+^ T cells. As mentioned earlier, 4-1BB costimulation triggers qualitatively distinct CD8^+^ T cells, characterized by enhanced effector memory differentiation. This type of memory response is known to exhibit rapid cytotoxic function that can quickly eliminate initial foci of infection, unlike other subsets that require a longer time to kill virally infected cells (27). Importantly, effector memory CD8^+^ T cells are positioned in blood and tissues (36), rendering them able to protect against systemic or mucosal challenges. Replicating cytomegalovirus (CMV) vectors trigger similar effector memory CD8^+^ T cell responses shown to protect against SIV infection in 50% of vaccinated macaques (37–40), but replicating vectors raise safety concerns, so elucidating alternative strategies to generate effector memory CD8^+^ T cells has been of interest in HIV vaccinology. As suggested by Masopust and Picker, vaccines against rapidly replicating intracellular pathogens (such as HIV, LCMV, or *Listeria*) are thought to necessitate effector memory CD8^+^ T cells to quickly control infection before the pathogen undergoes exponential replication (41). The observation that 4-1BB costimulation elicits effector and effector memory CD8^+^ T cells could prove useful for an HIV vaccine, because these subsets are especially poised for rapid killing of virally infected cells at frontline tissues. Overall, enhancing CD8^+^ T cells by α4-1BB may translate into better protection from symptomatic SARS-CoV-2 disease, considering their established role in reducing disease severity by eliminating infected cells and not necessarily by preventing initial infection, as that would be a function of antibodies (6, 42).

The classical 2-signal model postulates that T cell activation is dependent on 2 concurrent signals, antigen recognition and costimulation, also known as Signal 1 and Signal 2, respectively (7). For decades, this model has been a blueprint for understanding how T cell responses are generated and has had broad implications for the development of immunotherapies and vaccine adjuvants aimed at triggering costimulation. However, it is currently believed that both signals must occur concurrently, exactly at the same time, otherwise the T cell would undergo anergy. Our data show potential benefits of delaying costimulation, but future studies are needed to determine the generalizability of these findings to other costimulatory pathways besides 4-1BB or OX40. Overall, these studies may be important for the development of more effective vaccines and for understanding the time-dependent effects of 4-1BB costimulation.

### Limitations of the study.

A potential limitation of our findings is the need for vaccinees to undergo an additional injection with costimulatory antibodies 4 days after initial vaccination. To address this logistical challenge, future studies will examine the effects of encapsulating costimulatory α4-1BB in slow-release formulations, which could be coadministered with the vaccine on the same day. Another consideration is that costimulatory α4-1BB can induce inflammation, which is a reason why costimulatory α4-1BB have not yet been licensed (25). However, in our studies we used a single low dose of costimulatory α4-1BB, and we showed that the effects on vaccine responses were similar to when we administered high, repetitive doses, suggesting that the single low dose goes a long way. We also did not observe an increase in ALT activity with our single low dose of α4-1BB relative to control, further suggesting that the single-low-dose regimen was safe, but further studies are needed to determine safety more rigorously.

## Methods

### Sex as a biological variable.

Our study examined male and female animals, and similar findings are reported for both sexes.

### Mice, vaccinations, and antibody treatments.

Six- to 8-week-old C57BL/6 mice were used. Mice were purchased from The Jackson Laboratory (approximately half males and half females). Mice were immunized intramuscularly with mRNA-LNPs (made in-house) or MVA vectors (from Bernard Moss, NIH, Bethesda, Maryland, USA) diluted in sterile PBS. Mice received agonistic α4-1BB (clone 3H3, BioXcell) or IgG control antibody (clone 2A3, BioXcell) intraperitoneally at 50 μg or 200 μg per mouse on the indicated days, diluted in sterile PBS. To block GM-CSF, we treated mice with an αGM-CSF blocking antibody (clone MP1-22E9, Leinco) intraperitoneally at 500 μg per mouse on days 4 and 7 after vaccination. We utilized OX40 costimulatory antibody clone OX-86 from Leinco Technologies Inc (catalog C855). Mice were housed at Northwestern University’s Center for Comparative Medicine (CCM) or University of Illinois at Chicago (UIC).

### Reagents, flow cytometry, and equipment.

Single-cell suspensions were obtained from PBMCs and various tissues. Dead cells were gated out using Live/Dead fixable dead cell stain (Invitrogen). SARS-CoV-2 spike and SF162 peptide pools were used for intracellular cytokine staining and these were obtained from BEI Resources. Biotinylated MHC class I monomers (K^b^VL8, sequence VNFNFNGL; D^b^GP33, sequence KAVYNFATC; D^b^GP276, sequence SGVENPGGYCL; OVA, sequence SIINFEKL; and K^b^B8R, sequence TSYKFESV) were used for detecting virus-specific CD8^+^ T cells, and were obtained from the NIH tetramer facility at Emory University. Cells were stained with fluorescently labeled antibodies against CD8α (clone 53-6.7; PerCP-Cy5.5), CD44 (clone IM7; FITC), CD62L (clone MEL-14; PE-Cy7), CD127 (clone A7R34; Pacific Blue), TNF-α (clone MP6-XT22; PE-Cy7), IFN-γ (clone XMG1.2; APC), or Ki67 (clone SolA15; PE-Cy7), or with APC-labeled tetramers described above. Fluorescently labeled antibodies were purchased from BD Pharmingen, except for anti-CD127 and anti-CD44, which were from BioLegend. Flow cytometry samples were acquired with a Becton Dickinson FACSCanto II or an LSRII and analyzed using FlowJo v10 (Tree Star).

### SARS-CoV-2 spike, SARS-CoV-1 spike, OVA, HIV (SF162) envelope, and MVA lysate-specific ELISA.

Binding antibody titers were measured using ELISA as described previously (4, 43–48). In brief, 96-well flat bottom plates MaxiSorp (Thermo Fisher Scientific) were coated with 0.1 μg/well of the respective spike protein, for 48 hours at 4°C. For detection of MVA-specific antibody responses, MVA lysates were used as coating antigen (incubated for 48 hours at room temperature). Plates were washed with PBS plus 0.05% Tween 20. Blocking was performed for 4 hours at room temperature with 200 μL of PBS with 0.05% Tween 20 and bovine serum albumin. Six microliters of sera were added to 144 μL of blocking solution in the first column of the plate, 1:3 serial dilutions were performed until row 12 for each sample, and plates were incubated for 60 minutes at room temperature. Plates were washed 3 times followed by the addition of horseradish peroxidase–conjugated goat anti–mouse IgG (Southern Biotech) diluted in blocking solution (1:5000), at 100 μL/well and incubated for 60 minutes at room temperature. Plates were washed 3 times and 100 μL/well of Sure Blue substrate (Sera Care) was added for approximately 8 minutes. The reaction was stopped using 100 μL/well of KPL TMB stop solution (Sera Care). Absorbance was measured at 450 nm using a Spectramax Plus 384 (Molecular Devices). SARS-CoV-2 spike protein was produced in-house using a plasmid produced under HHSN272201400008C and obtained from BEI Resources, National Institute of Allergy and Infectious Diseases (NIAID), NIH: vector pCAGGS containing the SARS-related coronavirus 2; Wuhan-Hu-1 spike glycoprotein gene (soluble, stabilized) (catalog NR-52394). SARS-CoV-1 spike protein was obtained through BEI Resources, NIAID, NIH: SARS-CoV Spike (S) Protein deltaTM, Recombinant from Baculovirus (catalog NR-722). OVA protein was purchased from Worthington Biochemical (catalog LS003049). HIV-SF162 protein was obtained through the NIH AIDS Reagent Program, Division of AIDS, NIAID, NIH: Human Immunodeficiency Virus Type 1 SF162 gp140 Trimer Protein, Recombinant from HEK293T Cells, ARP-12026, contributed by Leo Stamatatos (Fred Hutchinson Cancer Center Seattle, Washington, USA).

### mRNA-LNP vaccines.

We synthesized mRNA vaccines encoding the codon-optimized SARS-CoV-2 spike protein from USA-WA1/2020, OC43 spike protein, OVA from the SERPINB14 gene, HIV-1 SF162 envelope protein, or the LCMV GP. Constructs were purchased from Integrated DNA Technologies or Genscript and contained a T7 promoter site for in vitro transcription of mRNA. The sequences of the 5′- and 3′-UTRs were identical to those used in a previous publication (44). All mRNAs were encapsulated into lipid nanoparticles using the NanoAssemblr Benchtop system (Precision NanoSystems) and confirmed to have similar encapsulation efficiency (~95%). mRNA was diluted in Formulation Buffer (catalog NWW0043, Precision NanoSystems) to 0.17 mg/mL and then run through a laminar flow cartridge with GenVoy ILM encapsulation lipids (catalog NWW0041, Precision NanoSystems) with N/P (lipid mix/mRNA ratio of 4) at a flow ratio of 3:1 (RNA: GenVoy-ILM), with a total flow rate of 12 mL/min, to produce mRNA-LNPs. mRNA-LNPs were evaluated for encapsulation efficiency and mRNA concentration using the RiboGreen assay and the Quant-iT RiboGreen RNA Assay Kit (catalog R11490, Invitrogen/Thermo Fisher Scientific).

### RNA-seq data acquisition and analysis.

C57BL/6 mice were immunized with 3 μg of mRNA-SARS-CoV-2 spike, and on day 4, treated with α4-1BB. On day 7, splenic CD8^+^ T cells were MACS-sorted with a MACS negative selection kit (STEMCELL Technologies). Purified CD8^+^ T cells were stained with K^b^VL8 tetramer, Live/Dead stain, and antibodies for CD8^+^ and CD44^+^ to gate on virus-specific CD8^+^ T cells. Live, CD8^+^CD44^+^K^b^VL8^+^ cells were FACS-purified to approximately 99% purity on a FACSAria cytometer (BD Biosciences) and delivered to Admera Health Biopharma for RNA extraction using Illumina 2 × 150 and RNA-seq using SMARTseq V4 with the NexteraXT kit. After the library was sequenced, the output file in BCL format was converted to FASTQ files and aligned to the mouse genome to generate a matrix file using the Cell Ranger pipeline (10X Genomics). These upstream QC steps were performed by Slim Fourati at Northwestern University, Chicago, Illinois, USA. Further analyses were performed in R using the Seurat package v4.0, as previously described (49). Terminal effector gene signatures were derived using the edgeR package (50), comparing effector memory to terminal effector CD8^+^ T cells (51). Clusters representing less than 4% of each population were excluded from downstream analyses.

### Adoptive transfer of P14 cells to measure expression kinetics of costimulatory molecules.

CD8^+^ T cells from Thy1.1^+^ P14 mice (PBMCs) were enriched using a CD8 MACS-negative selection kit (STEMCELL Technologies). Approximately 40,000 P14 CD8^+^ T cells were transferred intravenously into naive Thy1.2^+^ C57BL/6 recipient mice. Recipient mice were vaccinated intramuscularly with 3 μg of the mRNA-LCMV vaccine 24 hours later. PBMCs were collected at various time points to measure 4-1BB expression on donor (Thy1.1^+^) P14 T cells by flow cytometry.

### Multiplex cytokine/chemokine assay.

Blood samples were centrifugated at 21,130*g* for 10 minutes at 4°C to separate the serum. The serum samples were collected and frozen at –80°C until used. A multiplex cytokines/chemokines kit was purchased from Mesoscale Diagnostics LLC and used for quantifying serum cytokines/chemokines.

### ALT assay.

To detect serum ALT activity, sera were obtained from vaccinated mice treated with α4-1BB or control antibodies. ALT activity was measured using a colorimetric ALT Assay kit (catalog MA-ALT, RayBiotech) following the manufacturer’s instructions.

### Challenge models.

LCMV Cl-13 stocks were expanded in Vero-E6 cells (catalog CRL-1586, ATCC), using a protocol from a prior paper (52). LCMV titers were determined by plaque assay on Vero-E6 cell monolayers. LCMV Cl-13 challenges were intravenously at 2 × 10^6^ PFU/mouse, and *Listeria* (LM-OVA) challenges were intravenously at 1 × 10^7^ CFU/mouse.

### LCMV quantification.

Seed stock of LCMV Cl-13 was obtained from Rafi Ahmed’s laboratory (Emory University, Atlanta, Georgia, USA). The virus was propagated and tittered on BHK21 cells (catalog CCL-10, ATCC). BHK21 cells were passaged in DMEM with 10% fetal bovine serum (FBS). Cells were inoculated with a low MOI (0.1) in 1% DMEM and incubated for 72 hours. Titers were determined by plaque assay on Vero-E6 cell monolayers. Sera and spleen were collected at various time points after challenge. Infectious viral titers were determined by plaque assay using Vero-E6 cells. Vero-E6 cells (5 × 10^5^ per well) were seeded in 6-well plates in 10% DMEM; the monolayer was 90%–100% confluent after 24 hours. The spleen was homogenized using a standard TissueRuptur homogenizer (Qiagen), and 10-fold serial dilutions of tissues were made and then transferred drop-wise onto the cell monolayer. Sera dilutions were created in 10% DMEM and added drop-wise on the cell monolayer. Six-well plates were placed in a 37°C 5% CO_2_ incubator for 1 hour and manually rocked every 10 minutes. A 1:1 agarose/2 × 199 media monolayer was dispensed after 1 hour incubation and plates were incubated at 37°C and 5% CO_2_ for 96 hours. After 96 hours, a 1:50 1% neutral red solution was added to a 1:1 agarose/2 × 199 media mixture and overlaid onto plates. Plaques were counted the following day after agar overlay removal.

### Listeria quantification.

Spleens were collected from infected mice on day 3 after challenge. Bacterial titers were quantified by homogenizing tissues through a 42-μm strainer and resuspended in 1% Triton X-100. Ten-fold serial dilutions were created in 1% Triton X-100 and added drop-wise onto 6-well BHK agar plates. Plates were manually rocked and then incubated at 37°C and 5% CO_2_ for 24 hours. Colonies were counted the next day.

### B16-OVA melanoma model to study therapeutic vaccination.

B16-OVA melanoma cells were a gift from Jennifer Wu (Northwestern University, Chicago, Illinois, USA). Mice were injected subcutaneously with 2 × 10^6^ B16-OVA tumor cells used in prior studies (53). On day 10 after tumor challenge, mice were vaccinated intramuscularly with 3 μg of mRNA-OVA, followed by treatment with 50 μg of control antibodies or α4-1BB at different time points. Tumor volume was calculated as length × width × width × 0.5.

### In vivo bioluminescence.

We utilized an mRNA-LNP expressing a luciferase reporter (mRNA-luc) to examine whether 4-1BB affected antigen (luciferase) levels following mRNA vaccination. To quantify luciferase expression, luciferin (catalog LUCK-100, GoldBio) was administered intraperitoneally 15 minutes before imaging, as described previously (45, 54). Mice were anesthetized and imaged using an SII Lago IVIS Imager (Spectral Instruments Imaging). Region of interest (ROI) bioluminescence was used to quantify the signal. Each leg (quadriceps) was plotted as an individual immunization site.

### Statistics.

Statistical tests used are indicated on each figure legend. Dashed lines in data figures represent the limit of detection. Data represent mean and error bars represent SEM. Statistical significance was established at a *P* value of 0.05 or less. Data were analyzed using Prism version 10 (GraphPad).

### Study approval.

Mouse studies were performed at Northwestern University following biosafety level 2 guidelines with approval of the Institutional Animal Care and Use Committee under protocols IS00003324, IS00029076, IS00015002, IS00008785, and IS00003258.

### Data availability.

RNA-seq data were deposited in the NCBI Gene Expression Omnibus (GEO) database under accession number GSE260817, at https://www.ncbi.nlm.nih.gov/geo/query/acc.cgi?acc=GSE260817 Other data are available upon request. Raw data associated with the main article and supplemental material are included in the Supporting Data Values file.

## Author contributions

PPM and SS designed the experiments. SS performed most of the experiments. TD, MHL, NI, and BA helped with some of the immunogenicity experiments. SF analyzed the gene expression data. PPM and SS wrote the manuscript with feedback from all authors.

## Supplementary Material

Supplemental data

Supporting data values

## Version 1 10/21/2024

In-Press Preview

## Version 2 12/16/2024

Electronic publication
