# Supplementary material for: Delayed reinforcement of costimulation improves the efficacy of mRNA vaccines in mice
Source: J Clin Invest. 2024 Oct 21;134(24):e183973. doi: 10.1172/JCI183973 (PMC11645141; doi:10.1172/JCI183973)
Supplement: Supplemental data [file jci-134-183973-s007.pdf]

**Delayed reinforcement of costimulation improves effectiveness of mRNA vaccines in mice**

Sarah Sanchez[1], Tanushree Dangi[1], Bakare Awakoaiye[1], Min Han Lew[1], Nahid Irani[1], Slim Fourati[2], Pablo Penaloza-MacMaster*[1]

[1]Department of Microbiology-Immunology, Feinberg School of Medicine, Northwestern University, Chicago, IL 60611, USA. [2]Department of Medicine, Division of Allergy and Immunology, Feinberg School of Medicine and Center for Human Immunobiology, Northwestern University, Chicago, IL 60611, USA.

***Correspondence:**

Pablo Penaloza-MacMaster (ppm@northwestern.edu)

303 E. Chicago Ave. Tarry 6-728, Chicago, IL 60611

Phone: (312) 503-0357

**Short Title:**

Delayed 4-1BB improves vaccines

**Supplemental Figures:**

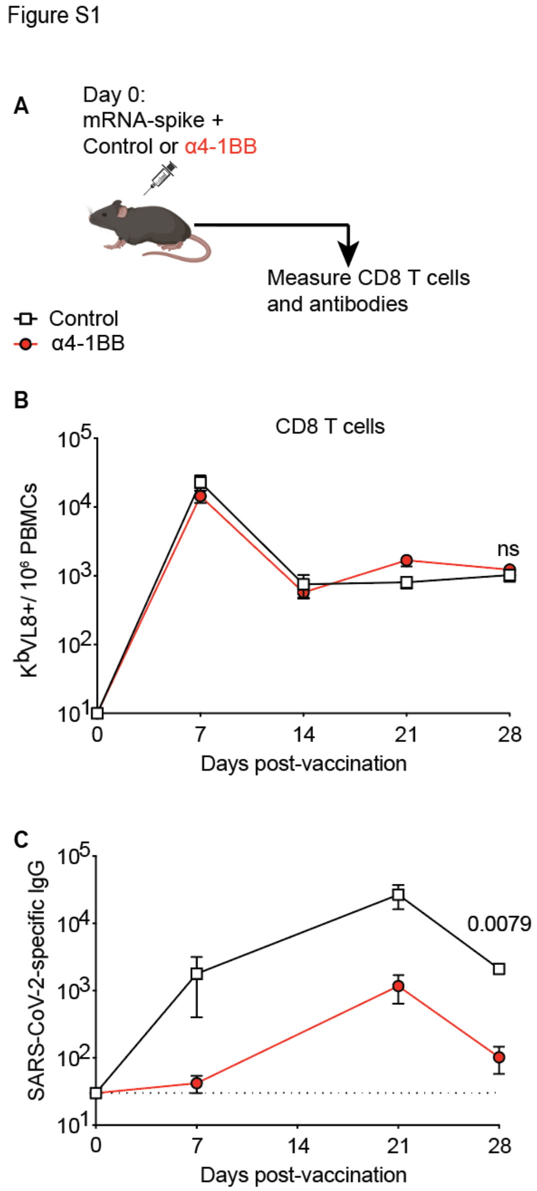


**Supplemental Figure 1. Reinforcing 4-1BB costimulation on the same day of vaccination does not improve immune responses.** (**A**) Experimental outline for evaluating the effect of α4-1BB at day 0 of vaccination. Mice were immunized with 3 μg of an mRNA-spike vaccine followed by treatment with 50 μg of α4-1BB or control antibodies on the same day. (**B**) Summary of virus-specific CD8 T cells. (**C**) Summary of antibody responses. Data are from one experiment, n=5 per group; experiment was performed twice with similar results. Indicated P values were calculated by the Mann–Whitney test at the last time point.

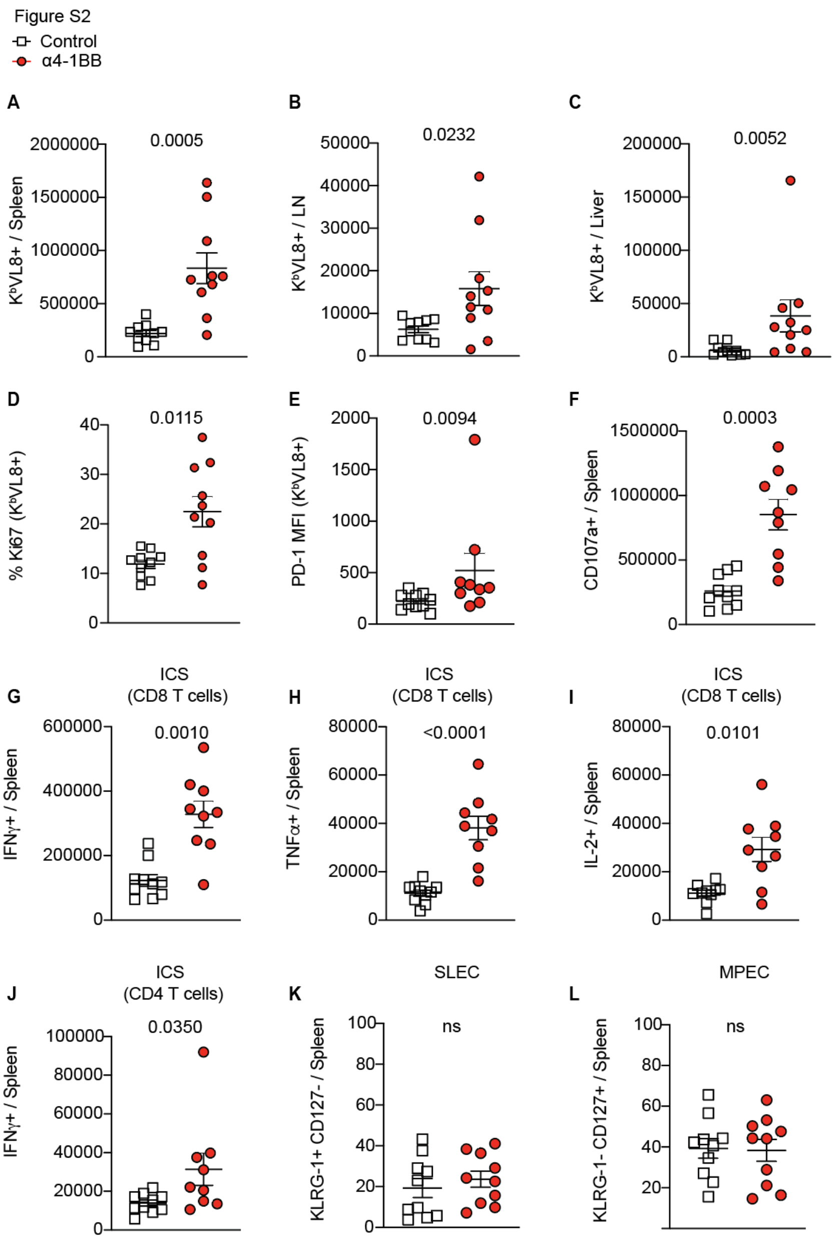


**Supplemental Figure 2. Virus-specific CD8 T cells in tissues.** Summary of CD8 T cell responses in spleen (**A**), lymph nodes (**B**), and liver (**C**) at day 7 post-vaccination. Summary of Ki67 (**D**) and PD-1 (**E**) expression on splenic CD8 T cells. Summary of CD107a (**F**), IFNγ (**G**), TNFα (**H**), IL-2 (**I**) on virus-specific CD8 T cells. Summary of IFNγ (**J**) on virus-specific CD4 T cells. Data from panels F-J are from intracellular cytokine staining (ICS) at day 15 post-vaccination, using spike overlapping peptide pools in the presence of GolgiPlug and GolgiStop. Summary of SLEC and MPEC subsets on splenic CD8 T cells ($K^b$VL8+) at day 30 post-vaccination. Mice were immunized with 3 µg of an mRNA-spike vaccine followed by treatment with 50 µg of α4-1BB or control antibodies. Data are from two experiments, n=5 per group/experiment. Indicated P values were calculated by the Mann–Whitney test.

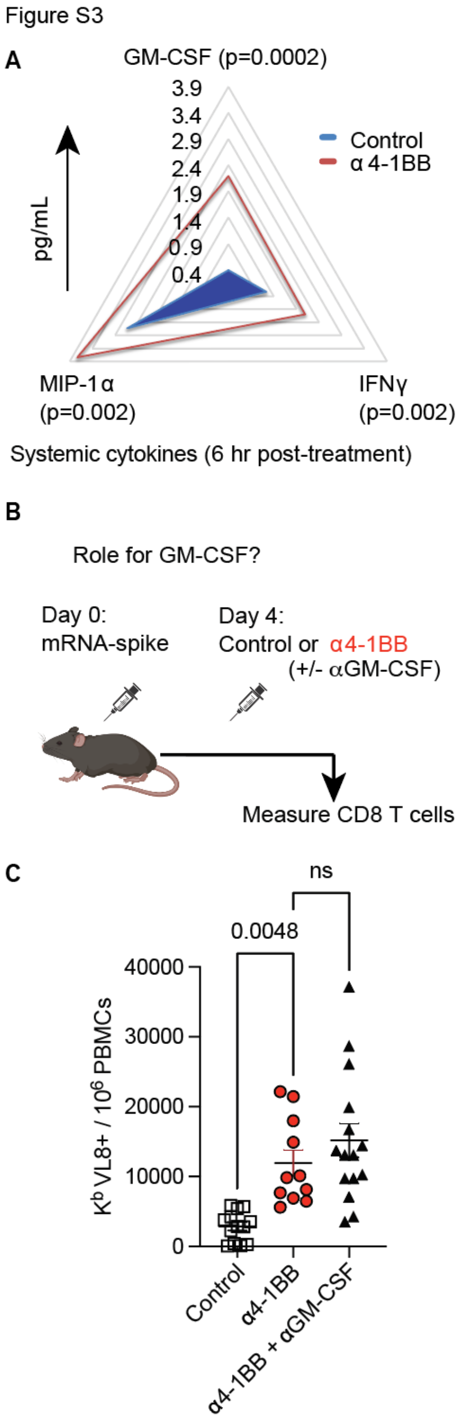


**Supplemental Figure 3. Systemic cytokines after treatment with 4-1BB costimulatory antibodies**. **(A)** Radar plots showing cytokines in serum 6 hr after treatment with 4-1BB costimulatory antibodies. 4-1BB costimulatory antibodies were administered at day 4 post-vaccination, same as in Figure 1C, and cytokines were quantified 6 hr after treatment. Data are from two experiments, n=5 per group/experiment. All data are shown. Indicated P values were calculated by the Mann–Whitney test. (**B**) Experimental outline for evaluating the mechanistic role of GM-CSF in the improvement of CD8 T cells following treatment with α4-1BB. (**C**) Summary of CD8 T cell responses in blood at day 14 post-vaccination. Data are from two experiments, n=3-5 per group/experiment. All data are shown. Indicated P values in panel C were calculated by one-way ANOVA (multiple comparisons).

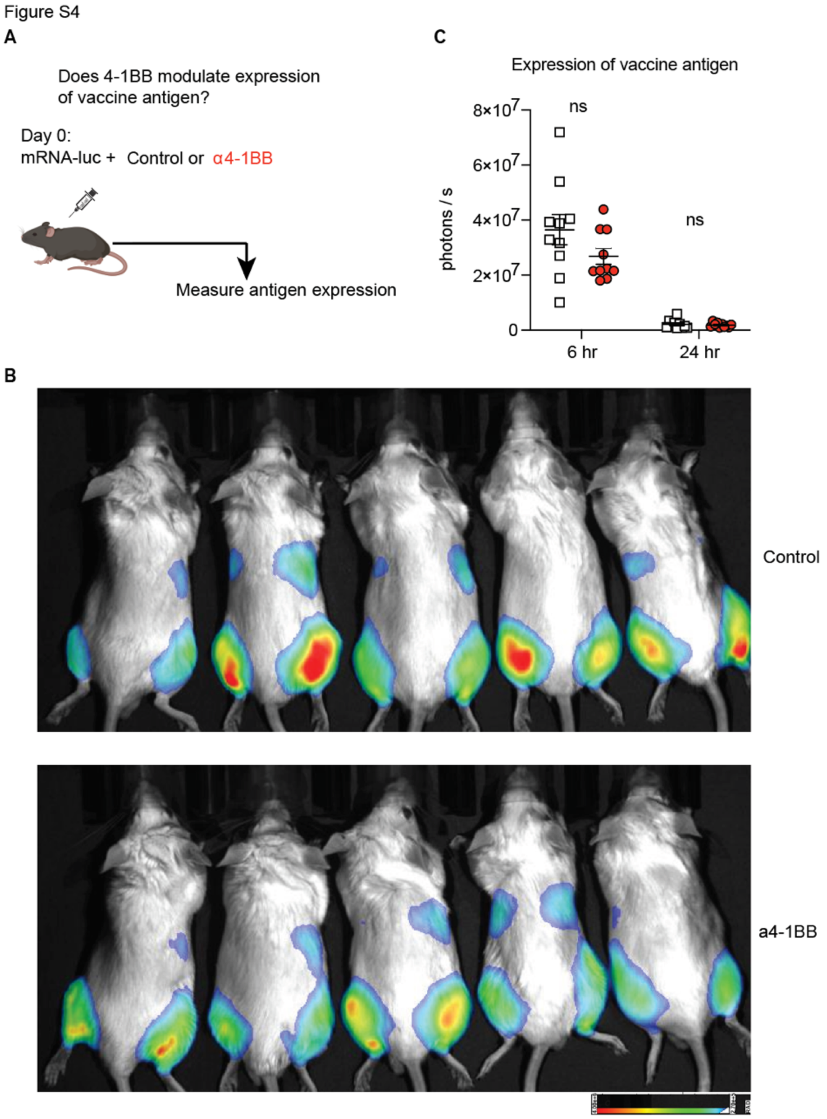


**Supplemental Figure 4. 4-1BB costimulation does not significantly affect antigen expression following mRNA vaccination.** **(A)** Experimental outline for quantifying antigen expression following reinforcement of 4-1BB costimulation. We utilized BALB/c mice, since their white coat facilitates visualization by bioluminescence. BALB/c mice were immunized intramuscularly with 3 µg of an mRNA-luciferase, and after 30 min, they were treated intraperitoneally with 50 µg of α4-1BB or control antibodies. After 6 hr post-immunization, mice were injected intraperitoneally with luciferin and luciferase expression was quantified by in vivo bioluminescence. (**B**) Bioluminescence images at 6 hr post-immunization. (**C**) Summary of antigen expression by bioluminescence. Data are from one experiment, n=5 per group/experiment (each quadriceps represents a separate immunization site, equating 10 quadriceps per group). All data are shown. Indicated P values were calculated by the Mann–Whitney test.

Figure S5

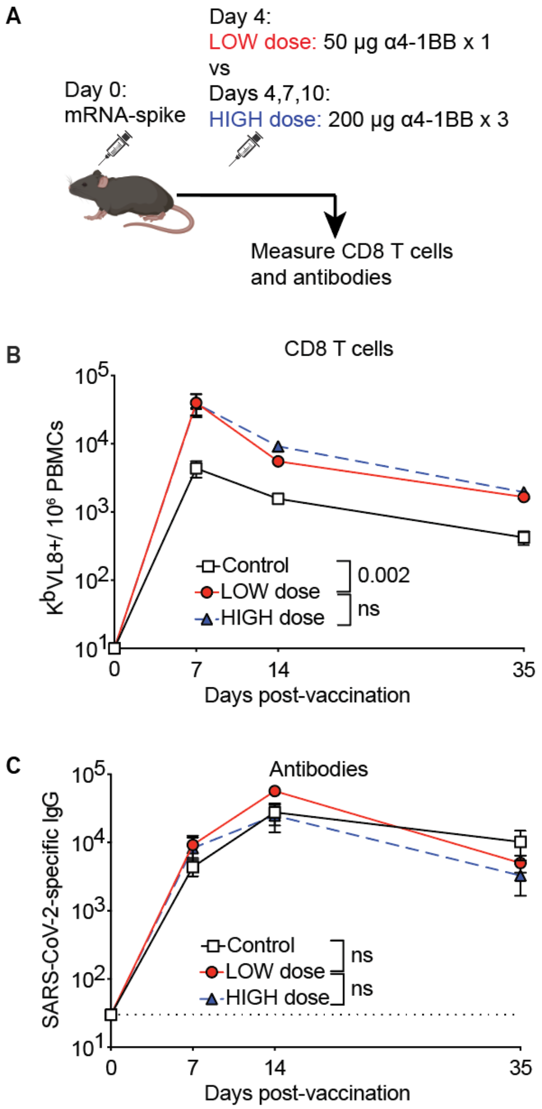


**Supplemental Figure 5. Continuously treating with α4-1BB after day 4 does not result in superior responses relative to treating just once at day 4**. (**A**) Experimental outline for comparing the effects of α4-1BB dose. Mice were vaccinated with 3 µg of an mRNA-spike vaccine. One group of mice received a single dose of 50 µg of α4-1BB on day 4 (**LOW dose**); another group of mice received 200 µg of α4-1BB on days 4, 7, and 10 (**HIGH dose**). (**B**) Summary of virus-specific CD8 T cell responses in PBMCs. (**C**) Summary of antibody responses in sera. Data are from one experiment with n=5 per group. Indicated P values were determined by 2-way ANOVA (Dunnett's multiple comparisons tests) at the last time point.

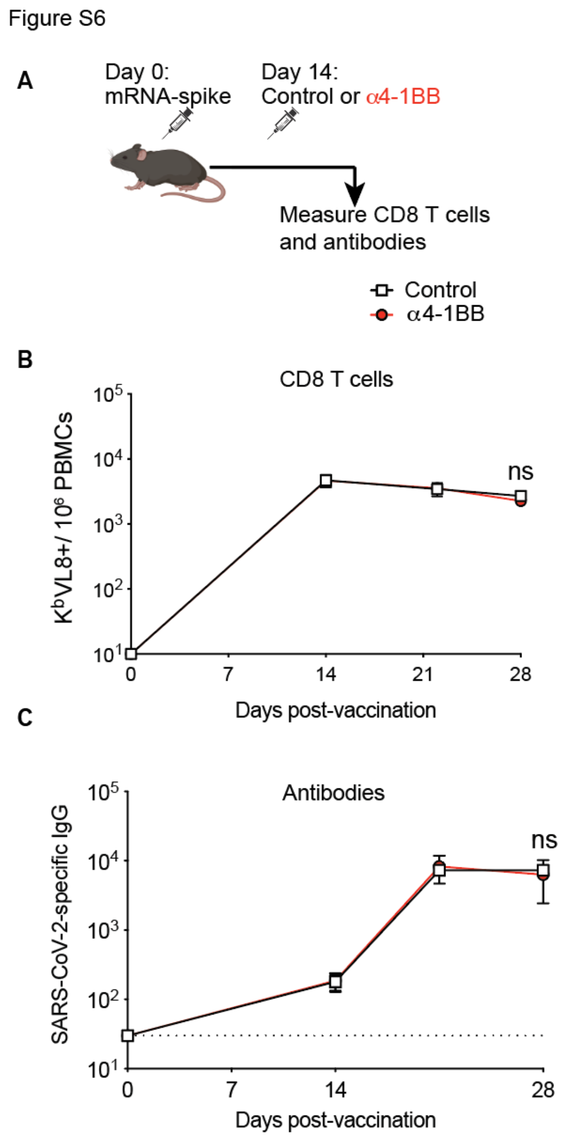


**Supplemental Figure 6. 4-1BB costimulation after day 14 does not result in improvement of immune responses following mRNA-SARS-CoV-2 vaccination. (A)** Experimental outline for evaluating whether treatment with α4-1BB after 2 weeks improves immune responses elicited by an mRNA-spike vaccine. C57BL/6 mice were immunized with 3 μg of an mRNA-spike vaccine followed by treatment with 50 μg of α4-1BB or control antibodies at day 14. **(B)** Summary of virus-specific CD8 T cell responses in PBMCs. (**C**) Summary of antibody responses in sera. Data are from one experiment with n=5 per group. Indicated P values were calculated by the Mann–Whitney test at the last time point.

Figure S7

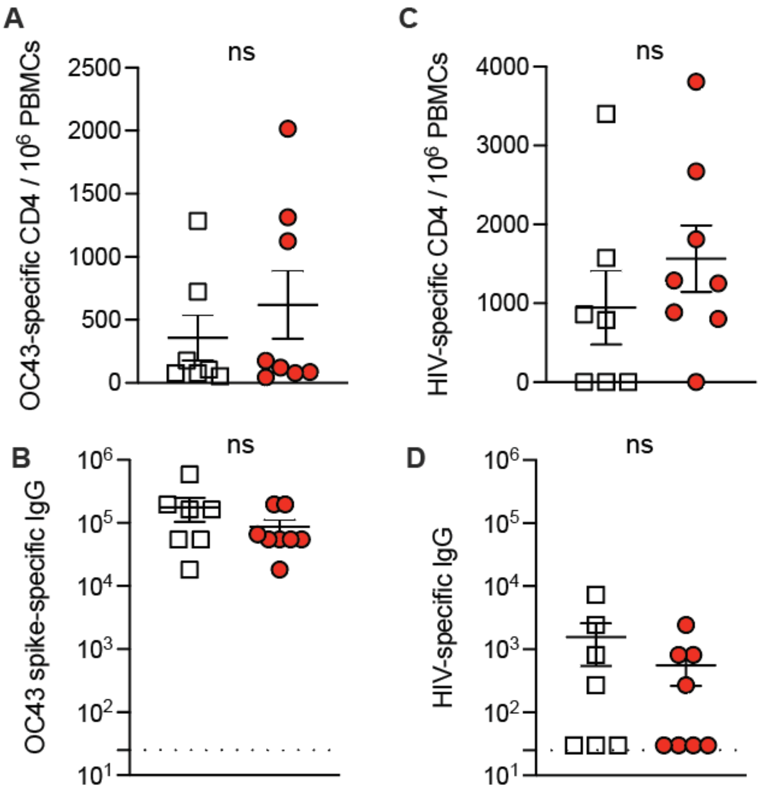


**Supplemental Figure 7. CD4 T cell and antibody responses are not significantly different following treatment with 4-1BB costimulatory antibodies at day 4. (A)** Summary of OC43 spike-specific CD4 T cell responses in PBMCs. **(B)** Summary of OC43 spike-specific antibody responses in sera. **(C)** Summary of HIV env-specific CD4 T cell responses in PBMCs. **(D)** Summary of HIV env-specific antibody responses in sera. Mice were immunized with 3 μg of each respective mRNA vaccine followed by treatment with 50 μg α4-1BB or control antibodies at day 4. Data from panels A and C are after intracellular cytokine stimulation using overlapping peptide pools (IFNγ+). Data are from day 14 post-vaccination. Data are from two experiments, one with n=5 per group/experiment and another one with n=2-3 per group/experiment. All data are shown. Indicated P values were calculated by the Mann–Whitney test.

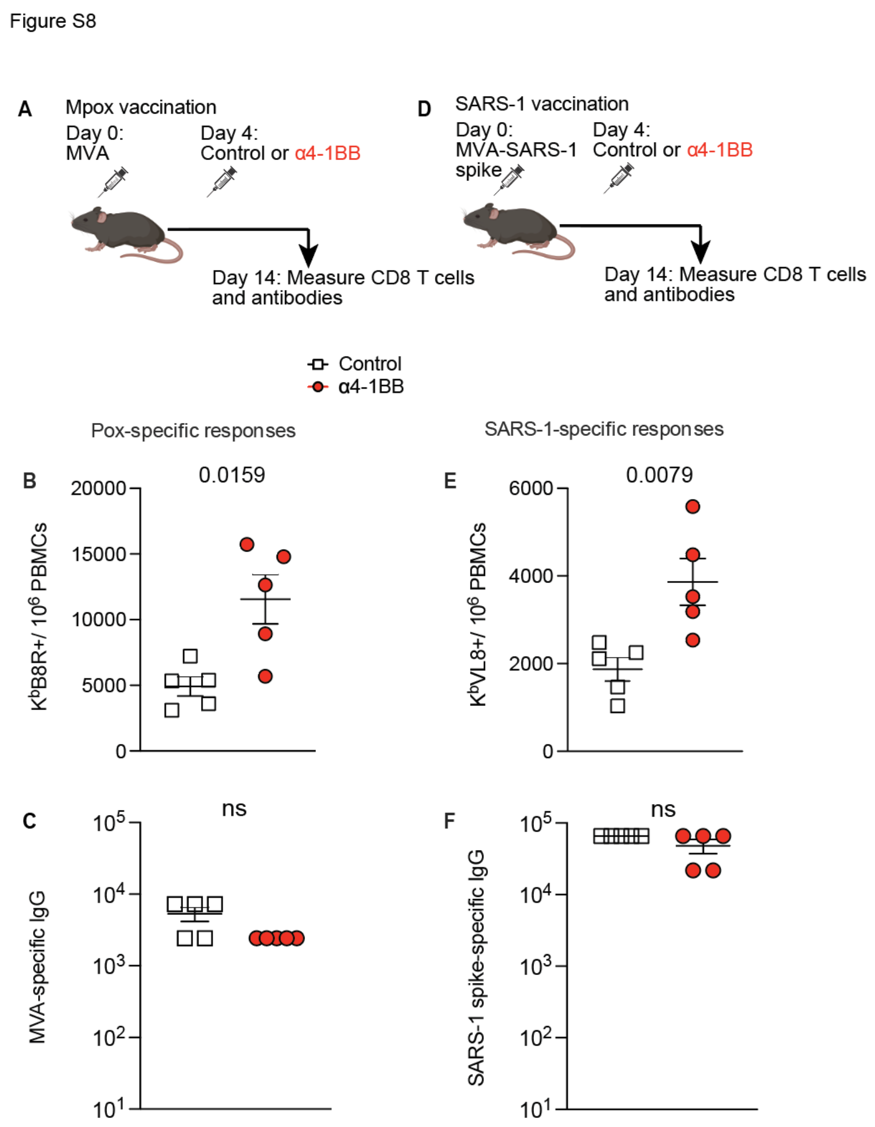


**Supplemental Figure 8. Generalizability to another vaccine platform: a poxvirus vector. (A)** Experimental outline for evaluating whether treatment with α4-1BB improves immune responses elicited by an MVA vaccine. **(B)** Summary of MVA-specific CD8 T cell responses in PBMCs. **(C)** Summary of MVA-specific antibody responses in sera. **(D)** Experimental outline for evaluating whether treatment with α4-1BB improves immune responses elicited by an MVA-SARS-CoV-1 vaccine. **(E)** Summary of SARS-CoV-1 specific CD8 T cell responses in PBMCs. **(F)** Summary of SARS-CoV-1-specific antibody responses in sera. Mice were vaccinated with $10^7$ PFU of the respective MVA vector followed by treatment with 50 µg of α4-1BB or control antibodies at day 4. Data are from day 14 post-vaccination. Data are from one experiment, n=5 per group. Indicated P values were calculated by the Mann–Whitney test.

Figure S9

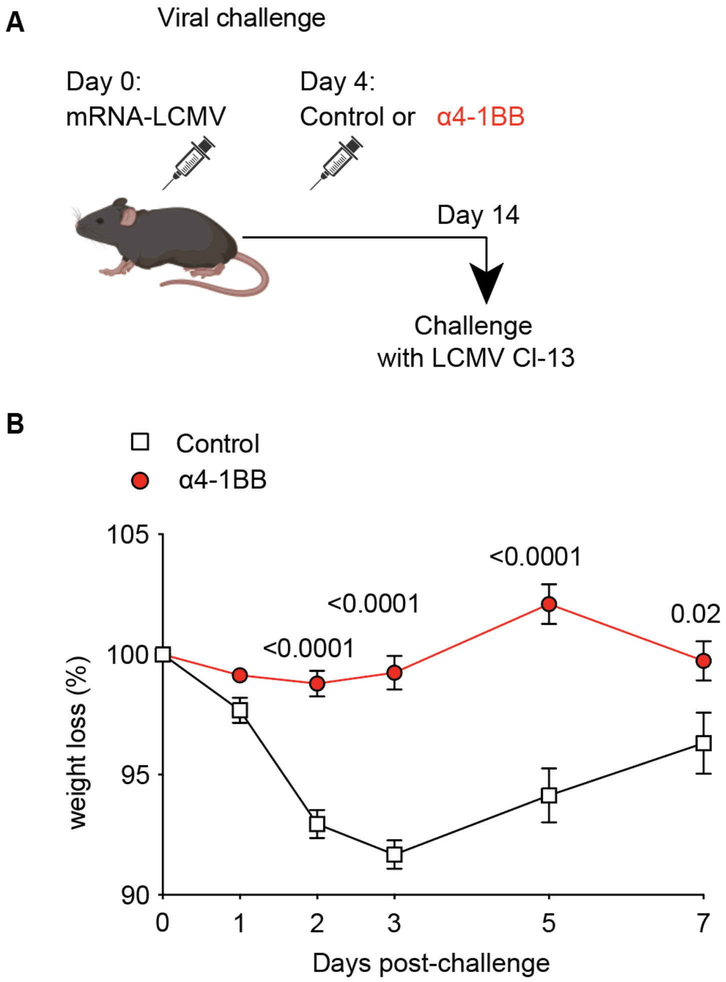


**Supplemental Figure 9. Reinforcing 4-1BB costimulation 4 days after mRNA-LCMV vaccination prevents weight loss after chronic LCMV Cl-13 challenge.** **(A)** Experimental outline to examine if treatment with α4-1BB at day 4 improves immune protection conferred by an mRNA-LCMV vaccine (same mice from Figure 4A-4B). **(B)** Summary of weight loss after LCMV Cl-13 challenge. Data are from two experiments, each with n=5 per group/experiment. Indicated P values were calculated by the Mann–Whitney test.

Figure S10

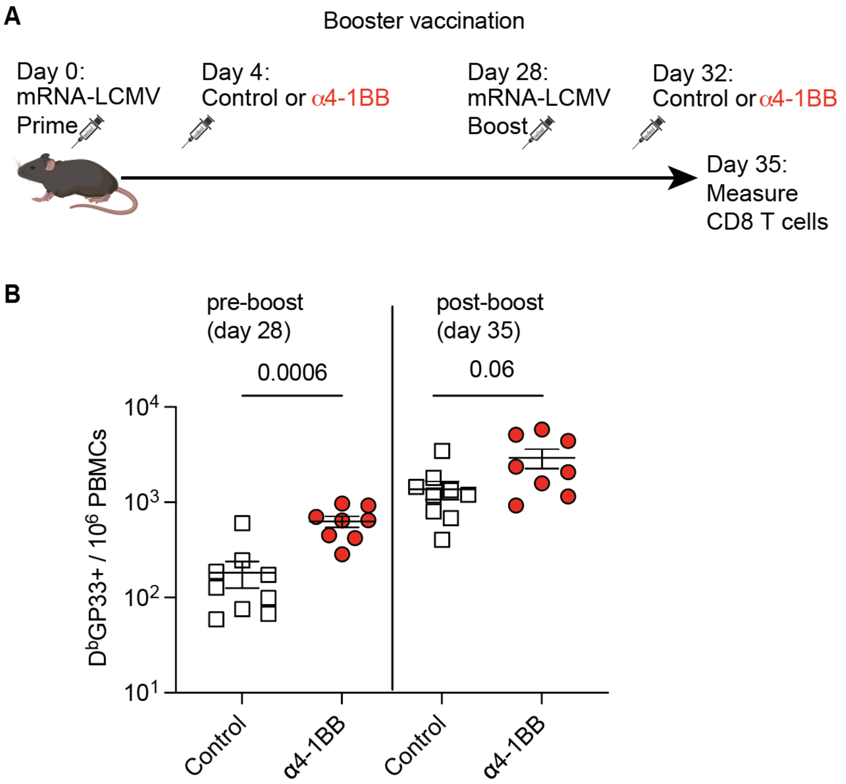


**Supplemental Figure 10. Effect of reinforcing 4-1BB costimulation in a prime-boost vaccine regimen. (A)** Experimental outline to examine the effects of α4-1BB during a recall response. Mice were primed with 3 µg of an mRNA-LCMV vaccine followed by treatment with 50 µg of α4-1BB or control antibodies at day 4 post-prime. At day 28 post-prime, mice were boosted homologously and treated with 50 µg of α4-1BB or control antibodies at day 4 post-boost. (**B**) Summary of LCMV-specific CD8 T cell responses in PBMCs. Data are from two experiments, with n=4-5 per group/experiment. All data are shown. Indicated P values were calculated by the Mann–Whitney test.

Figure S11

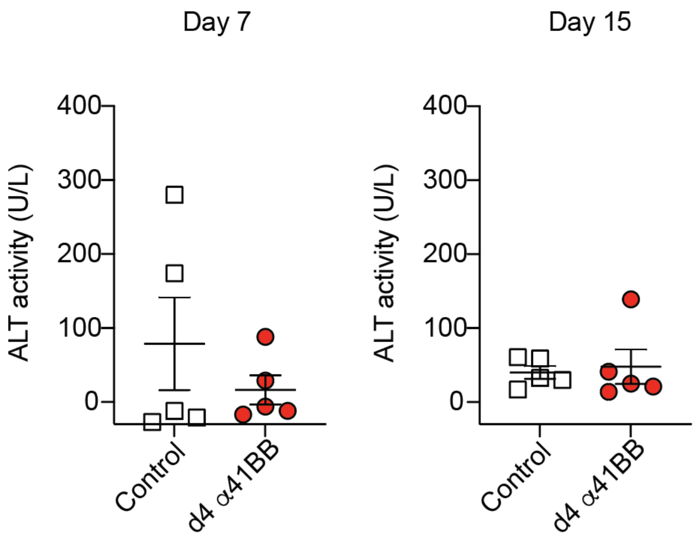


**Supplemental Figure 11. Treatment with α4-1BB antibody at day 4 post-vaccination does not increase alanine aminotransferase (ALT) activity in sera relative to control vaccinated mice**. Mice were immunized with 3 µg of mRNA-spike vaccine followed by treatment with a single dose of α4-1BB (50 µg) or control antibodies at day 4. ALT activity was quantified in sera after vaccination. Data are from one experiment, with n=5 per group. Indicated P values were calculated using the Mann–Whitney test.

Figure S12

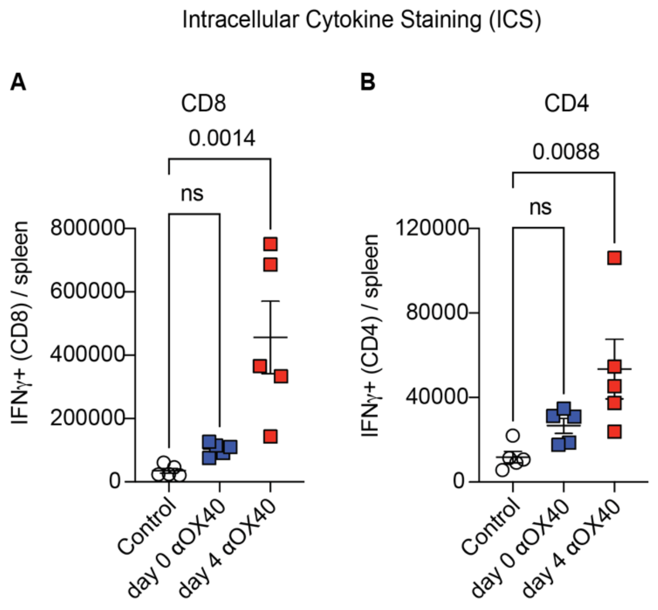


**Supplemental Figure 12. Cytokine expression on virus-specific CD8 and CD4 T cells after reinforcing OX40 costimulation.** The experimental outline was identical to that of Figure 6C. Splenic CD8 T cells (**A**) and CD4 T cells (**B**) responses at day 30 post-vaccination are shown. Data are after intracellular cytokine stimulation using overlapping peptide pools (IFNγ+). Data are from one experiment, with n=5 per group. Indicated P values were calculated by the Mann–Whitney test.

Figure S13

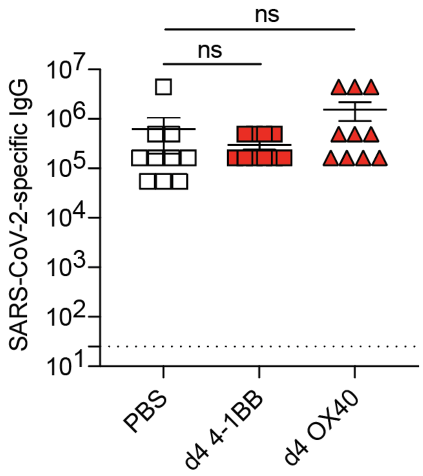


**Supplemental Figure 13. Comparative analyses of antibody responses following α4-1BB or αOX40 treatment.** The experimental outline was identical to that of Figs. 1A and 6C. Antibody responses in sera at day 15 post-vaccination are shown. Data are from two experiments, with n=5 per group. Indicated P values were calculated by the Kruskal Wallis test (Dunn's multiple comparisons).
